# Comparison of Rooting Strategies to Explore Rock Fractures for Shallow Soil-Adapted Tree Species with Contrasting Aboveground Growth Rates: A Greenhouse Microcosm Experiment

**DOI:** 10.3389/fpls.2017.01651

**Published:** 2017-09-22

**Authors:** Yunpeng Nie, Hongsong Chen, Yali Ding, Jing Yang, Kelin Wang

**Affiliations:** ^1^Key Laboratory of Agro-ecological Processes in Subtropical Region, Institute of Subtropical Agriculture, Chinese Academy of Sciences Changsha, China; ^2^Huanjiang Observation and Research Station for Karst Ecosystems, Chinese Academy of Sciences Huanjiang, China; ^3^University of Chinese Academy of Sciences Beijing, China; ^4^College of Forestry, Guizhou University Guiyang, China

**Keywords:** weathered bedrock, drought stress, physiological response, root biomass allocation, drought adaptation, karst region of southwest China

## Abstract

For tree species adapted to shallow soil environments, rooting strategies that efficiently explore rock fractures are important because soil water depletion occurs frequently. However, two questions: (a) to what extent shallow soil-adapted species rely on exploring rock fractures and (b) what outcomes result from drought stress, have rarely been tested. Therefore, based on the expectation that early development of roots into deep soil layers is at the cost of aboveground growth, seedlings of three tree species (*Cyclobalanopsis glauca, Delavaya toxocarpa*, and *Acer cinnamomifolium*) with distinct aboveground growth rates were selected from a typical shallow soil region. In a greenhouse experiment that mimics the basic features of shallow soil environments, 1-year-old seedlings were transplanted into simulated microcosms of shallow soil overlaying fractured bedrock. Root biomass allocation and leaf physiological activities, as well as leaf δ^13^C values were investigated and compared for two treatments: regular irrigation and repeated cycles of drought stress. Our results show that the three species differed in their rooting strategies in the context of encountering rock fractures, however, these strategies were not closely related to the aboveground growth rate. For the slowest-growing seedling, *C. glauca*, percentages of root mass in the fractures, as well as in the soil layer between soil and bedrock increased significantly under both treatments, indicating a specialized rooting strategy that facilitated the exploration of rock fractures. Early investment in deep root growth was likely critical to the establishment of this drought-vulnerable species. For the intermediate-growing, *A. cinnamomifolium*, percentages of root mass in the bedrock and interface soil layers were relatively low and exhibited no obvious change under either treatment. This limited need to explore rock fractures was compensated by a conservative water use strategy. For the fast-growing, *D. toxocarpa*, percentages of root mass in the bedrock and interface layers increased simultaneously under drought conditions, but not under irrigated conditions. This drought-induced rooting plasticity was associated with drought avoidance by this species. Although, root development might have been affected by the simulated microcosm, contrasting results among the three species indicated that efficient use of rock fractures is not a necessary or specialized strategy of shallow-soil adapted species. The establishment and persistence of these species relied on the mutual complementation between their species-specific rooting strategies and drought adaptations.

## Introduction

Large portions of the world’s terrestrial landscapes are characterized by shallow soils overlying bedrock (Schwinning, 2010). In contrast to a rooting medium composed of homogenous soil, where a developing root system can spread freely in all directions, the rooting medium of shallow soil regions is generally characterized by highly constrained root growth especially in the vertical direction (Goodsheller, 2010). Additionally, a series of early studies conducted in shallow soil regions reported that roots of many species could grow through connected cracks and fissures into deep weathered bedrock layers (Wang et al., 1995; Zwieniecki and Newton, 1995; Hubbert et al., 2001; Witty et al., 2003). More recently, studies in southwest Australia further reported that seedlings of shallow-soil endemics employed specialized root strategies not shared by congeners living on nearby deeper soils (Poot and Lambers, 2008; Poot et al., 2012). As roots encountering layers of bedrock or other obstacles commonly end abruptly and/or are replaced by horizontal extensions (Porterfield, 2002; Baluška et al., 2009), the unusual increase in root biomass close to the bedrock surface was treated as a specialized strategy increasing the chance to locate downward conduits into deeper layers (Poot and Lambers, 2008; Schwinning, 2013). This idea is creative and the related results provide insight into the rooting strategies of shallow-soil adapted species. However, a lack of research on comparable shallow-soil systems currently limits this generalization.

Karst regions are typical shallow soil regions and occupy 10–15% of the total continental area (Yuan, 1994; Ford and Williams, 2013). Most weathered materials that are derived from the soluble carbonated bedrock are removed by water flow and result in shallow soil coverage (Cao et al., 2005). Additionally, shallow soils are usually underlain by weathered bedrock, which is manifested by networks of fractures and fissures (Bonacci et al., 2009). The combination of shallow soil and permeable bedrock normally results in rapid hydrological processes and an unfavorable soil-water environment for plants (Butscher and Huggenberger, 2009). A series of studies provided evidence that species in karst regions accessed water in bedrock layers through their extensive root systems (Jackson et al., 1999; Querejeta et al., 2007; Estrada-Medina et al., 2013). On the contrary, other studies suggested that plant roots were mainly restricted to the shallow soil layer (Heilman et al., 2009; Kukowski et al., 2013; Dammeyer et al., 2016). These contrasting results presumably indicate that shallow-soil adapted species employ different rooting strategies in regard to exploring rock fractures. However, the probable existence of diverse rooting strategies remains to be explored.

The karst region of southwest China, for example, is one of the most typical landscapes developed on carbonate bedrock in the world (Yuan, 1994). Unlike most other karst regions, this area receives a large amount of annual precipitation (normally ranging between 1000 and 2000 mm; Jiang et al., 2014). The precipitation in this area also shows clear seasonal variation, with more than 70% of the precipitation occurring between May and September (Chen et al., 2011). These complex water conditions, including an abundant and temporally concentrated distribution of precipitation, which contributes to the diversification of plant water use strategies (Bonetti et al., 2017), and high heterogeneous water storage capacity of karst substrate (Tokumoto et al., 2014), are likely to cause diverse water use and rooting strategies in karst species (Fan et al., 2011). Therefore, the karst region of southwest China is an ideal platform on which to investigate rooting strategies employed by shallow-soil adapted species.

Root-related features of karst species in southwest China have long been the main focus of research in this area, however, very limited progress has been achieved because of the harsh habitat which made belowground observations very difficult (Weaver and Jurena, 2009). Among them, studies aimed at revealing plant biomass allocation found that karst vegetation has a higher ratio of root to aboveground biomass than non-karst vegetation in the same bioclimatic zone (Luo et al., 2010; Ni et al., 2015). Our previous study found that coarse root systems of two widely distributed karst species were dominated by horizontal extension rather than deep penetration in different shallow-soil habitats (Nie et al., 2014a). However, none of the previously mentioned authors continued to study the related rooting strategies, which could help provide insight into their findings. Previous studies have demonstrated the substantial effects of water dynamic (such as repeated cycles of drought stress) on plant root development at fine time scales (Woods et al., 2011; Padilla et al., 2013). As the frequency (as well as the duration time) of consecutive days without rain in southwest China is increased under the circumstance of climate change (Liu et al., 2015), it is reasonable and necessary to conduct related studies on fine time scale water dynamics. Although there are studies on the response of plant water use to repeated cycles of dry-down and rewatering (Wei et al., 2007; Liu et al., 2010), very few of them were conducted for the purpose of uncovering plant-rooting strategies.

The study area experienced severe deforestation from 1958 to the mid-1980s due to human disturbances (e.g., agriculture expansion, fuelwood collection and livestock overgrazing), which resulted in severe soil erosion and, as a consequence, the area was left with shallow, rocky residual soil and sparse vegetation cover (Chen et al., 2011). In order to mitigate the impacts of the degraded environment, actions were carried out to promote forest restoration under the national policies controlling payments for ecosystem services (Liu et al., 2008; Jiang et al., 2014). Great effort was put into the establishment of plantations and species were selected based on their adaptions to shallow-soil environments (Mei et al., 2004). This selection of plantation species provides an ideal background to conduct comparative studies among different shallow-soil adapted species. Furthermore, investigation of rooting strategies that are employed by different species in this area is of practical importance for selecting appropriate species for forest restoration.

As exploration of rock fractures by plants is generally at the cost of reduced energy allocation to aboveground biomass (Poorter and Nagel, 2000), rooting strategies of plants are supposed to be closely related to their aboveground growth rates. In the current study, tree species with distinct seedling growth rates were selected from the species pool used in karst reforestation in subtropical China. In a greenhouse experiment, 1-year-old seedlings were transplanted into microcosms of shallow soil underlain by an artificial rock layer with simulated fractures. After growing for 2 months, seedlings were exposed to either regular irrigation or repeated cycles of drought stress. Both root biomass allocation and leaf physiological activities were investigated to test the hypotheses that (1) species with higher aboveground growth rates are weaker in their rock fracture exploration capacity, and (2) repeated cycles of drought stress can enhance root deployment in rock fractures.

## Materials and Methods

### Species Selection

Three tree species, *Cyclobalanopsis glauc*a, *Delavaya toxocarpa*, and *Acer cinnamomifolium* were selected among those recommended for forest restoration in the karst region of southwest China. All three species are native to the area and can be found naturally growing on karst hillslopes and, thus, are treated as shallow soil-adapted species. Moreover, seedlings of these three species exhibit differences in aboveground growth rates, which is thought to be related to their diverse rooting strategies. Among them, the evergreen species, *C. glauca*, is the slowest growing. It is widely distributed in subtropical Asia and regarded as a species that prefers shallow soil and a calcium-rich environment (Lou and Zhou, 2001). In the karst region of South China, *C. glauca* is mostly found in late successional communities (such as secondary forest). The deciduous species, *D. toxocarpa*, is the fastest-growing among the selected species. This species is found across a wide climate range (from the hot subtropics to the cool temperate zone) and is regarded as a generalist species with respect to soil moisture conditions (Lv et al., 2009). The third species, *A. cinnamomifolium*, is evergreen and has an intermediate growth rate. This species can be found in several non-arid provinces of China and is known to prefer moist environments (Qin and Liu, 2010).

### Experimental Design

In preparation for the experiment, seeds were collected from karst hillslopes (limestone) around Guilin City (110°10′-110°429′E, 24°40′-25°40′N), Guangxi Province, Southwest China. A subtropical monsoon climate dominates this region, with mean annual rainfall around 1,900 mm and mean annual temperature around 19.3°C. Seeds were germinated and seedlings were nursery grown in 1.18 L cylindrical containers (diameter = 10 cm; height = 15 cm). One-year-old seedlings were transported to the greenhouse at Huanjiang Observation and Research Station for Karst Ecosystems of the Chinese Academy of Sciences (108°18′E, 24°43′N) during early April 2013, where they were immediately transplanted into the prepared microcosms.

**FIGURE 1:**
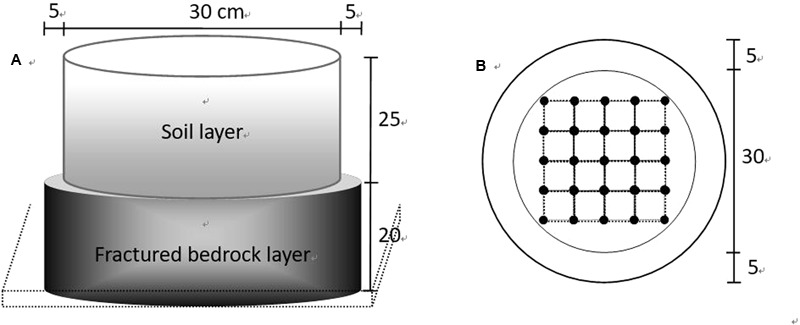
Sketch of the experimental plot that was used to simulate local substrate conditions (shallow soil over fractured bedrock). **(A)** A 25 cm height cylinder (30 cm in diameter, made from a section of PVC pipe without a bottom) was located in the middle-top of another 20 cm height cylinder (40 cm in diameter, made from concrete with designed fractures). **(B)** Fractures were represented by vertically distributed small cylinder cavities (2 cm in basal diameter) throughout the concrete body. Cavities (25 in total per cylinder) were evenly located within the inner circle (30 cm in diameter), indicated by the intersections of the grid.

The microcosms were designed to simulate field conditions with a thin soil layer over fractured bedrock (**Figure 1A**). The artificial bedrock layer was made from concrete, with individual cylinders having a diameter of 40 cm and a height of 20 cm. Fractures were represented by cylindrical holes (2 cm in diameter), vertically throughout the concrete. Twenty-five holes were evenly distributed within the inner circle of 30 cm in diameter and occupied 10% of the inner volume (**Figure 1B**). In total, 150 fractured concrete blocks were linearly placed in 12 shallow trenches (5 cm in depth and 50 cm in width) with 50 and 100 cm gaps between blocks and trenches, respectively. In order to guarantee that there was no water gathered at the bottom of the trenches during irrigation, the bottoms were gently sloped (<5°) by excavation and covered by waterproof film and 2 cm depth of fine sand.

Soil collected from the upper 20 cm depth on nearby karst hillslopes was prepared (air dried and well mixed) for the greenhouse experiment. According to the USDA classification, these were clay loam soils with soil organic matter, total nitrogen, total phosphorus and total potassium at 52.6, 5.1, 1.0 and 3.5 g⋅kg^-1^, respectively. In order to simulate the filled fractures in the field, all 25 holes in each concrete block were filled with soil by hand. Soil in the fractures was slightly compacted to achieve natural bulk densities (1.3 g cm^-3^, Yang et al., 2016). Then a 25 cm length PVC pipe (30 cm in diameter, opaque pipe) was located on the top middle of the concrete to simulate the shallow soil layer. The bottom 10 cm depth of the PVC pipe was filled with soil; seedlings were then transplanted into the upper 15 cm depth with one individual per microcosm. The bulk density in the PVC pipe was made similar to that in the field (1.1 g cm^-3^, Fu et al., 2015). One hundred and fifty seedlings (50 individuals per species) in total were transplanted into the microcosm systems along the 12 trenches (6 had 12 individuals, the others 13). Seedlings for two of the three different species were randomly assigned to each trench (6 or 7 individuals per species), and each trench was randomly assigned to a treatment (drought or well-watered).

Initially, seedlings were left to acclimate to the microcosms for approximately 2 months, during which, seedlings were liberally irrigated in the evening from the tops of the microcosms every 3 days. Fifteen individuals of each species were harvested at the end of the acclimation period on June 1st, 2013 to establish pre-treatment baseline values for each species. Two days later, 15 individuals per species were marked as control and irrigated regularly as before. In order to reveal the physiological response of species to changing water status, the rest of the 20 individuals of each species were exposed to dry-down and rewatering cycles. Specifically, irrigation from the top of the microcosm was completely stopped during each dry-down period. While, the thin sand layer beneath the microcosms was carefully irrigated to keep the sand humidity around field capacity throughout each dry-down period. It was assumed that soil in the simulated rock fractures (at least in the lower layers) could benefit from this humid sand layer largely based on soil water suction, thus maintaining a relatively stable water status. Wilted leaves were once found on *D. toxocarpa* (which exhibits the fastest growth rate and highest transpiration rate). The leaves did not recover on the morning of the following day. All drought treated individuals of the three species were rewatered from the tops of the microcosms every other day until no further improvement of photosynthesis occurred over two measuring days. The 3 month long experiment included three drought phases and two recovery periods; with the duration of each drought phase between 16 and 24 days and that of the two recovery phases were 18 and 20 days, respectively.

### Measurements

Leaf gas exchange was first measured on June 3rd, and then measured every 3 days throughout the experimental period. On each measuring day, three control and five drought treated individuals were randomly selected, and three newly formed mature leaves were measured for each individual. Leaf photosynthetic rate (A_n_) and stomatal conductance (g_s_) were measured using a portable open gas exchange system (Li-6400, LI-COR, Inc., Lincoln, NE, United States) between 8:30 and 11:30 am. For most of the measurements, the ambient relative humidity ranged from 40 to 66%, the vapor pressure deficit ranged between 1.3 to 2.9 kPa, and the temperature ranged between 27 to 33°C. Photosynthetic Photon Flux Density (PPFD) was set at 1200 μmol m^-2^ s^-1^ (a light-saturating level for all three species) using a standard leaf chamber equipped with a blue-red light source. Gas flow rate was set at 400 μmol s^-1^.

Leaf stable carbon isotope composition (δ^13^C) has long been used as a proxy for water use efficiency (WUE) of C_3_ plants based on a positive correlation between leaf δ^13^C value and WUE (Francey and Farquhar, 1982; Marshall and Zhang, 1994; Hasselquist et al., 2010). In the current study, leaf samples for stable carbon isotope analysis were collected separately at the beginning and the end of the experiment. Newly formed mature leaves were collected from 3 and 5 individuals per species and mixed as 1 sample (three replications per species per treatment). Leaf samples were oven dried at 70°C for 48 h, then frozen in liquid N_2_ and ground to a powder to pass an 80 mesh screen for δ^13^C analysis. Samples were measured using an isotopic ratio mass spectrometer (Finnigan MAT Delta V advantage, Thermo Finnigan, San Jose, CA, United States) at the Key Laboratory of Agro-ecological Processes in Subtropical Region, of the Chinese Academy of Sciences. The δ^13^C value was calculated as

(1) $$\delta^{13}\text{C}(\text{‰})\;=\;({\text{R}}_{\text{sample}}/{\text{R}}_{\text{standard}}-1)\;\times \;1000$$

where R_sample_ and R_standard_ are the ^13^C/^12^C ratios in the sample and in the conventional Pee Dee Belemnite standard, respectively.

Plant biomass allocation was determined at the beginning (15 individuals per species) and at the end of the experiment (35 individuals per species). The above-ground portion of the biomass was removed first and weighed after oven drying at 70°C for 48 h. Roots were harvested in three fractions: the upper 20 cm and the bottom 5 cm of the soil and roots occupying the cavities in the concrete (including roots in the bottom sand layer). The PVC pot containing the topsoil was first split vertically into two halves by using a portable electric saw. A big garden scissor was then used to cut the soil column at 20 cm depth and at the bottom, horizontally. Materials (root and soil) in the concrete cavities were carefully pushed out with a rubber stick. Roots were washed on a 1 mm sieve to remove the attached soils, and oven dried at 70°C for 48 h before weighing.

One-year-old seedlings were separately transplanted into microcosms, which were designed to simulate shallow soil above fractured bedrock. Microcosm-acclimated seedlings were exposed to either regular irrigation or repeated cycles of dry-down and rewatering. Moreover, the bottom of the fractured bedrock layer was kept humid throughout each dry-down period, thus, providing a different water status for the soil and the bedrock layers. Physiological responses of seedlings to changing water status were monitored throughout the experimental period. Samples of plant biomass and leaf δ^13^C values were collected at the beginning and the end of the experiment, respectively.

### Statistical Analysis

For the physiological data, one-way ANOVA was used to detect difference between treatments at each measurement date. Plant biomass data at different category levels and leaf δ^13^C values were first subjected to two-way analysis of variance (ANOVA) to determine the effects of treatment, species and their interaction at the end of the experiment. A Tukey’s HSD multiple comparison test was performed when significant differences were detected among species. Moreover, the differences among baseline and the two treatments for each species were further detected by one-way ANOVA. Non-normal distributed data were log or arcsin square root (for % values) transformed to meet the assumptions of ANOVA. All statistical analyses were performed with SPSS software (SPSS, Chicago, IL, United States).

## Results

### Physiological Activities under Different Water Treatments

**FIGURE 2:**
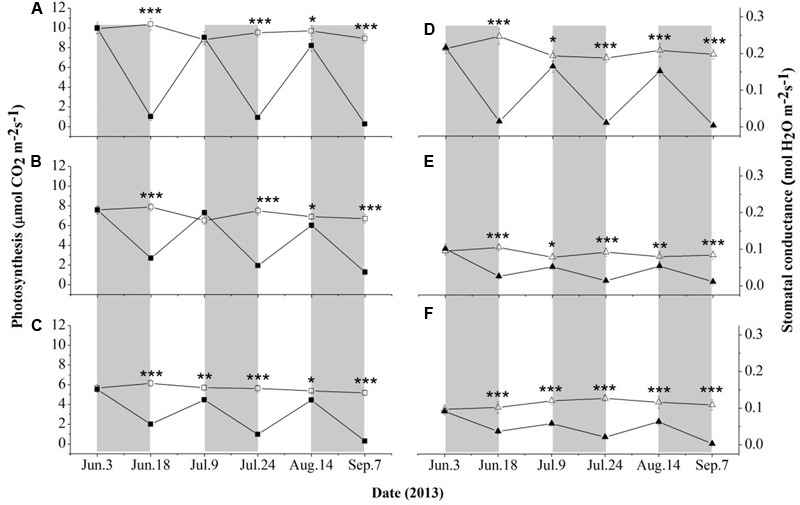
Time course of leaf photosynthesis and stomatal conductance for seedlings of three shallow soil-adapted tree species, *Delavaya toxocarpa* **(A,D)**, *Acer cinnamomifolium* **(B,E)** and *Cyclobalanopsis glauc*a **(C,F)**, growing under simulated local substrate conditions. *Error bars* represent ± 1 SE (*n* = 3 and 5 for the well-watered and drought treatments, respectively). Filled and open symbols represent the drought and well-watered treatments, respectively. The gray bars and the interspersed white zones indicate dry-down and rewatering phases during the drought treatment, respectively. Significant differences between treatments at each measuring date are: ^∗^*P* < 0.05; ^∗∗^*P* < 0.01, ^∗∗∗^*P* < 0.001.

As shown in **Figure 2**, seedlings of the selected three species exhibited relatively steady A_n_ and g_s_ under well-watered conditions throughout the study period. The values of A_n_ and g_s_ were significantly higher (*P* < 0.01) in *D. toxocarpa* than in *A. cinnamomifolium* and *C. glauc*a. *A. cinnamomifolium* also exhibited relatively higher values of A_n_ (*P* < 0.05) than *C. glauc*a; however, no significant difference was detected between their g_s_.

During the three drought phases, all three species showed rapid declines in A_n_ and g_s_ (**Figure 2**). The values of A_n_ and g_s_ in *D. toxocarpa* decreased by 92 and 95% on average, respectively, which were significantly higher (*P* < 0.05) than those of *A. cinnamomifolium* (72 and 78%) but not *C. glauc*a (79 and 81%; **Figure 2**). More specifically, *A. cinnamomifolium* (exhibiting intermediate growth rate) always maintained relatively higher (*P* < 0.05) values of A_n_ at the end of each drought phase than the other two species (**Figures 2A–C**).

At the end of the first rewatering phase, the values of A_n_ totally recovered for *D. toxocarpa* and *A. cinnamomifolium* but not for *C. glauca*, while none of them completely recovered at the end of the second phase (**Figures 2A–C**). None of the three species exhibited complete recovery of g_s_ during either rewatering phase (**Figures 2D–F**). Specifically, there was approximately 80% recovery of g_s_ in *D. toxocarpa* and *A. cinnamomifolium* at the end of the rewatering phases, which was significantly higher (*P* < 0.01) than that of *C. glauca* at 62%.

### Overall Effects of Treatment, Species and Their Interactions on Plant Biomass and Leaf δ^13^C Value

Both treatment and species had significant effects (*P* < 0.001) on above-ground and below-ground dry mass, as well as on total dry mass (**Table 1**). However, the interaction of these two factors only had significant effect (*P* < 0.05) on above-ground dry mass. On the one hand, only species but not treatment and its interaction with species had significant effect (*P* < 0.01) on the proportion of root mass. On the other hand, both of these two factors and their interactions had significant effects (at different levels) on percentage of roots in rock fractures and interface layer (**Table 1**). Moreover, both species (*P* < 0.001) and treatment (*P* = 0.043) but not their interactions had (marginally) significant effects on leaf δ^13^C value.

**Table 1:** Effects of treatment (T), species (S) and the interaction of these two factors (T × S) at the end of the experiment on leaf δ^13^C value, proportion of root mass, percentage of roots in rock fractures and roots in the bottom 5 cm soil (interface) layer.

	Factors	*F*-value	*P*-value		Factors	*F*-value	*P*-value
Above-ground dry mass (g)	T	90.976	<0.001^∗∗∗^	Percentage of roots in rock fractures (%)	T	17.614	<0.001^∗∗∗^
	S	379.651	<0.001^∗∗∗^		S	312.956	<0.001^∗∗∗^
	T × S	6.009	0.004^∗∗^		T × S	9.957	<0.001^∗∗∗^
Below-ground dry mass (g)	T	27.193	<0.001^∗∗∗^	Percentage of roots in interface layer (%)	T	4.463	0.037^∗^
	S	150.709	<0.001^∗∗∗^		S	403.084	<0.001^∗∗∗^
	T × S	2.229	0.114		T × S	7.887	0.001^∗∗^
Total dry mass (g)	T	98.434	<0.001^∗∗∗^	Leaf δ^13^C value (aaa)	T	5.112	0.043^∗^
	S	632.882	<0.001^∗∗∗^		S	16.762	<0.001^∗∗∗^
	T × S	2.882	0.062		T × S	2.017	0.176
Proportion of root mass (%)	T	2.258	0.137				
	S	5.776	0.004^∗∗^				
	T × S	2.614	0.079				

*^∗^*p* < 0.05, ^∗∗^*p* < 0.01, ^∗∗∗^*p* < 0.001.*

### Effects of Treatment on Leaf δ^13^C Values of Different Species

**FIGURE 3:**
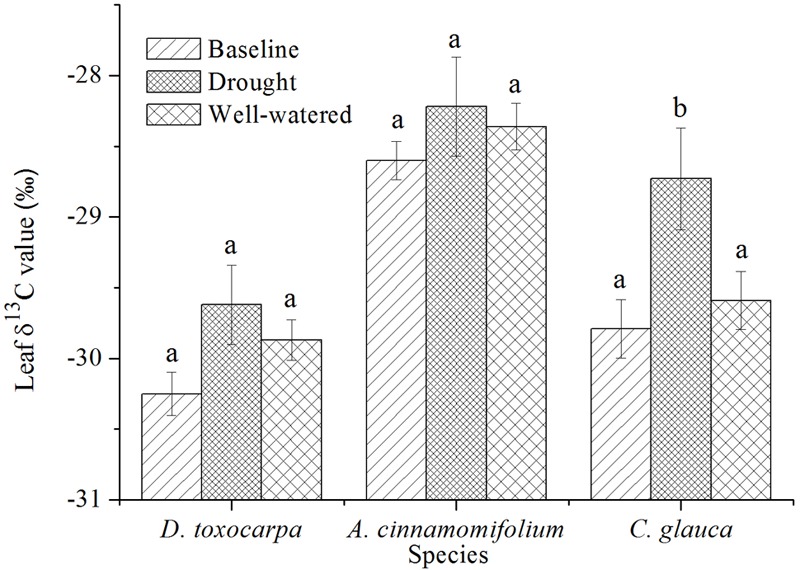
Comparison of mean leaf δ^13^C values for three seedling species growing under simulated local substrate conditions (shallow soil underlain by fractured bedrock). ‘Baseline*’* indicates values obtained after 2 months’ acclimation in the simulated substrate, whereas ‘drought*’* and ‘control*’* refer to values obtained at the end of the drought and well-watered treatments, respectively. *Error bars* represent ± 1 SE (*n* = 3; note that each replication is a bulk sample containing 3–5 seedlings). Different lowercase letters represent significant differences between means at *P* < 0.05.

As shown in **Figure 3**, there were no significant changes (compared to values for the initial harvest, the baseline) of leaf δ^13^C values for all three species under the well-watered treatment. Only *C. glauca* experienced a significant increase of leaf δ^13^C value under the drought treatment, which was also significantly higher than under the well-watered condition. Moreover, *A. cinnamomifolium* had consistently higher leaf δ^13^C values than the other species.

### Effects of Treatment on Plant Biomass Allocation

Compared to the initial harvest (the baseline), all species experienced significant increases in dry mass under both drought and well-watered treatments (**Table 2**). Within species, most dry mass values under well-watered conditions were significantly higher than those under the drought treatment. Moreover, *C. glauca* always had relatively higher root mass ratios even without an obvious increase under the drought treatment (**Table 2**).

**Table 2:** Biomass allocation for seedlings of different shallow soil-adapted species growing under simulated local substrate conditions.

	*D. toxocarpa*			*A. cinnamomifolium*			*C. glauca*		
	Baseline	Drought	Control	Baseline	Drought	Control	Baseline	Drought	Control
Above-ground dry mass (g)	17.4 ± 1.6a	23.8 ± 2.3b	29.6 ± 1.9c	13.2 ± 1.7a	18.4 ± 2.4b	21.1 ± 2.0b	7.2 ± 1.2a	10.1 ± 1.7b	12.7 ± 1.5b
Below-ground dry mass (g)	8.2 ± 0.9a	13.6 ± 1.6b	14.7 ± 1.3b	7.2 ± 0.9a	9.5 ± 1.4b	12.1 ± 1.4c	4.5 ± 0.7a	6.7 ± 1.1ab	7.9 ± 1.2b
Total dry mass (g)	25.6 ± 2.1a	37.4 ± 3.3b	44.3 ± 2.7c	20.4 ± 2.1a	27.9 ± 3.6b	33.2 ± 3.0b	11.7 ± 1.4a	16.8 ± 2.2b	20.6 ± 2.3c
Proportion of root mass (%)	0.32 ± 0.02a	0.36 ± 0.04a	0.33 ± 0.02a	0.35 ± 0.03a	0.34 ± 0.03a	0.36 ± 0.02a	0.39 ± 0.01ab	0.40 ± 0.03b	0.38 ± 0.02a

*‘Baseline’ indicates values obtained after 2 months’ acclimation in the simulated local substrate, whereas ‘drought’ and ‘control’ refer to the data obtained at the end of the experiment for the repeated cycles of drought stress and well-watered treatments, respectively (Mean ± 1 = SE; *n* = 15 for both baseline and control, 20 for the drought treatment). One-way ANOVA were used to identify all possible significant differences among all three categories. Different lowercase letters represent significant different between means of different categories of the same species at *P* < 0.05.*

**FIGURE 4:**
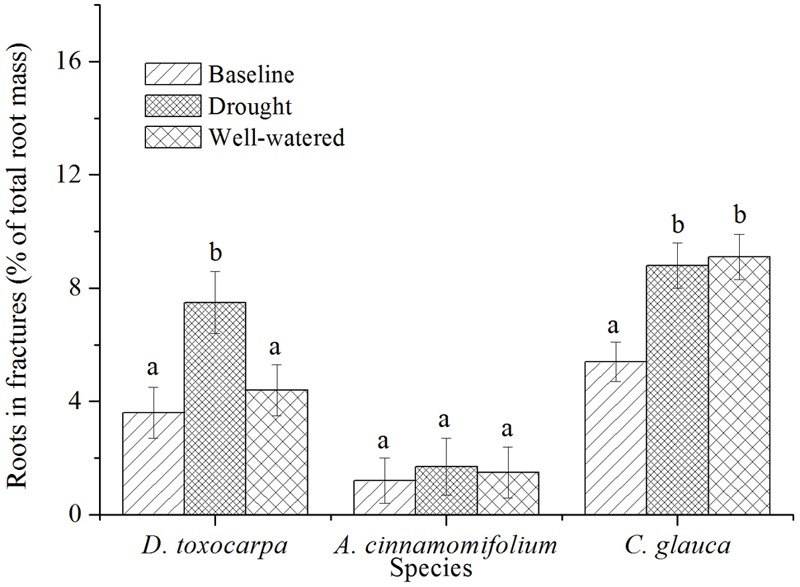
Mean percentage of root biomass found in the fractured bedrock layer for seedlings growing under simulated local substrate conditions. *Error bars* represent ± 1 SE (*n* = 15 for both baseline and control, 20 for the drought treatment). Interpretation of symbols are explained in the legend of **Figure 3**.

In order to determine how efficiently a species can locate artificial fractures, spatial placement of roots under different conditions was investigated. As shown in **Figure 4**, *A. cinnamomifolium* always placed a significantly lower percentage of roots in fissures than the other two species, with no obvious response to different treatments. Compared with the initial harvest, percentage of root allocation to rock fractures was significantly increased only under drought conditions in *D. toxocarpa*, whereas it was increased in both treatments in *C. glauca*.

**FIGURE 5:**
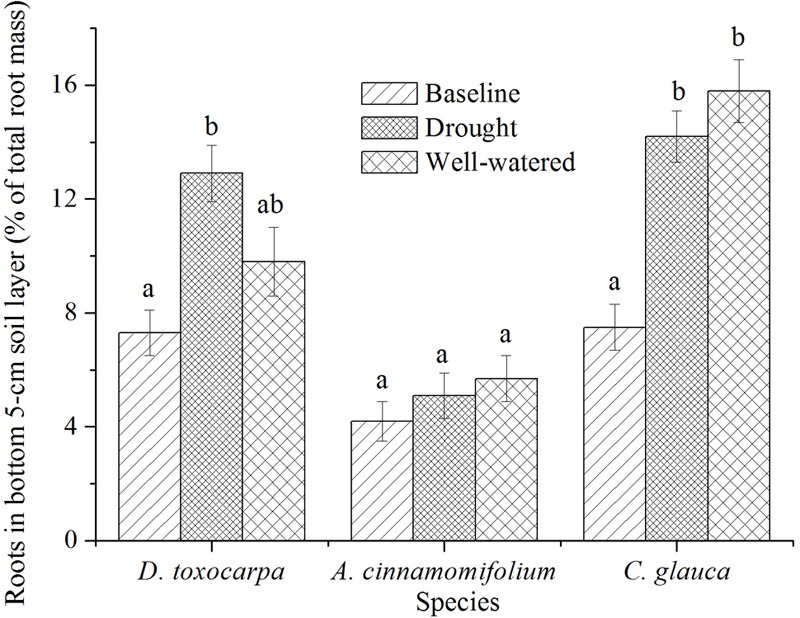
Mean percentage of root biomass found in the bottom 5 cm of soil for seedlings growing under simulated local substrate conditions. *Error bars* represent ± 1 SE (*n* = 15 for both baseline and control, 20 for the drought treatment). Interpretation of symbols are explained in the legend of **Figure 3**.

*Acer cinnamomifolium* seedlings exhibited relatively low and steady percentages of roots in the bottom 5-cm soil layer (the interface between the soil and the fractured layers, **Figure 5**). In contrast, both *D. toxocarpa* and *C. glauca* seedlings placed relatively high percentages of roots in this interface layer and showed an increased allocation over the course of the experiment for both treatments, with *C. glauca* seedlings having the highest allocation especially in the well-watered condition (**Figure 5**).

## Discussion

In the current study, microcosms were set up to simulate the growth environment for plant species established in karst regions. There is no doubt that field conditions could be much more complicated than the microcosm, and seedling growth might also be affected by the designed environment (Poorter et al., 2012). Thus, these results do not represent the performance of seedlings under field conditions because of the absence of field measurements (Kawaletz et al., 2014). On the other hand, the contrasting exploration of soil volume at the top of the bedrock layer provides an opportunity to reveal plant-rooting strategies in the context of encountering fractured obstacles. Although, we cannot exclude the possibility that root growth into the bedrock layer by chance can also result in the increase of root biomass in the fractures, a simultaneous increase of root biomass near the surface of the bedrock layer can be treated as a specific strategy which increases the chance to find underlying fractures (Poot and Lambers, 2008; Schwinning, 2013).

### Relationship between Seedling Growth Rate Aboveground and Root Exploration of Rock Fractures

For seedlings of the evergreen tree species, *C. glauca*, percentages of roots increased significantly not only in the fractures, but also in the interface layer under well-watered conditions, indicating that they employed a specialized rooting strategy, as described above (Poot and Lambers, 2008; Poot et al., 2012). In accordance with this speculation, previous studies in the same area found that sources of water used by adult individuals in the field usually decoupled from soil water (Huang et al., 2009; Deng et al., 2012; Gu et al., 2015). Studies in other karst regions further suggested that early development of roots into deeper layers where substantial water depletion does not occur could facilitate the establishment and growth of young trees (Jackson et al., 1999; Querejeta et al., 2007; Hasselquist et al., 2010). Besides, it was claimed that the exploration of fractured layers, to some extent, was less about resource capture and more about space pre-emption (Matthes and Larson, 2006; Schwinning, 2010). As seedlings of *C. glauca* exhibited the slowest aboveground growth rate among the selected three species, root performance of this species is in line with the first hypothesis that exploration of rock fractures is at the cost of aboveground growth. However, this hypothesis was not supported by the other two species.

Although seedlings of *A. cinnamomifolium* exhibited a medium growth rate, they did not allocate much root mass to the interface layer, and the percentage of roots in fractures was also relatively low under well-watered conditions. Moreover, root mass ratio and root biomass allocation exhibited no obvious changes under drought treatment. These results suggest that seedlings of *A. cinnamomifolium* employed relatively shallow root systems that do not actively forage for cracks in underlying rock. Root growth of most plants is thought to be inhibited when encountering physical barriers and the underlying mechanism may be related to self-inhibition of growth in response to an accumulation of root exudates close to a barrier (Falik et al., 2005). In this situation, roots usually change direction to grow toward areas of less resistance, or divert resource to increase growth of other laterals (Semchenko et al., 2008). Based on field excavation, our previous study conducted in the same region, also revealed the shallow root systems of adult individuals of other tree species (Nie et al., 2014a). Moreover, species with shallow root systems are generally associated with other features, such as a more sensitive response to small rain events and higher resource absorption/use efficiency (Pregitzer et al., 2002; Hultine et al., 2003), which may facilitate their establishment and persistence in this humid karst region.

Root deployment of fast-growing *D. toxocarpa* was largely influenced by the water conditions of the topsoil, which is different from the other two species. Compared with well-watered conditions, percentage of roots in rock fractures increased significantly under drought treatment, which was accompanied by a trend of increase (although not significant) of roots in the interface layer, suggesting a drought-induced fracture exploration strategy. From this point of view, the second hypothesis of this study can only be supported by the fast-growing species. It has been widely demonstrated that too much investment in deep roots, regardless of soil water status, by plants would inevitably reduce aboveground growth and competitiveness (Lavergne et al., 2004; Puri et al., 2015), thus minimizes the establishment and survival in habitats with intense aboveground competition. In line with this speculation, more specific studies suggest that, compared with narrow endemic species, widespread species were normally more tolerant to edaphic variation with higher adaptive plasticity (Bastida et al., 2014; Hand et al., 2017). Field investigations in karst regions of South China have demonstrated that communities of *D. toxocarpa* could be found in a variety of soil depths (Lv et al., 2009). The success of *D. toxocarpa* in different habitats might benefit from the pronounced root plasticity.

### Contrasting Strategies of Tree Species to Cope with Drought in Shallow Soils

Physiological responses to the repeated cycles of drought stress suggest that *C. glauca* was a more drought-vulnerable species than the other two species. Although all of three species exhibited continuously decreasing photosynthesis within each dry-down period, *C. glauca* showed the worst recovery of photosynthesis after the first rewatering. As stomatal closure is usually the main protective mechanism against drought (Flexas et al., 2009), recovery of photosynthesis is likely to have been limited by decreased stomatal conductance. Similar results have also been obtained on seedlings of other native species in karst regions of South China (Wei et al., 2007; Liu et al., 2010). Arguably, the limited recovery of photosynthesis under drought stress, followed by rewatering, necessitates an early development of roots into deeper layers, thus minimize the effects of drought at different time scales on plant growth.

Seedlings of *A. cinnamomifolium* maintained relatively low g_s_ even under well-watered conditions, which is indicative of a conservative water use strategy. Moreover, as carbon isotope composition of leaf tissue (represented by leaf δ^13^C value) has long been accepted as a proxy for intrinsic WUE of C3 plants (Francey and Farquhar, 1982), the relatively steady and significantly higher leaf δ^13^C values than found in the other two species indicate a relatively high WUE. Although, not focused on the same three species, our previous study also revealed contrasting leaf δ^13^C values among coexisting species on karst rocky outcrops (typical examples of shallow soil habitat; Nie et al., 2014b). In accordance with this water use strategy, seedlings of *A. cinnamomifolium* always maintained significantly higher A_n_, than the other two species, at the end of each drought phase. Besides, although the sizes of *A. cinnamomifolium* were not the smallest among the selected three species, both A_n_ and g_s_ of the drought-exposed seedlings were totally or largely recovered after rewatering. With its shallow root system, it is likely that *A. cinnamomifolium* relied on a water use strategy that made full use of the water in the shallow soil layer.

Physiological responses of *D. toxocarpa* to drought and rewatering indicated that it adopts a strategy of drought escape (Delzon, 2015). During drought phases, the values of A_n_ in *D. toxocarpa* decrease sharply and reach very low values at the end of each phase. Moreover, A_n_ completely or largely recovers following rewatering, which is accompanied by a good recovery of g_s_. This rapid recovery of leaf gas exchange compensated for the depression of carbon assimilation during drought periods (Liu et al., 2010). Furthermore, seedlings of *D. toxocarpa* were observed to start losing their fully expanded leaves (personal observation) when the newly formed leaves were still active during drought periods. For deciduous tree species, losing a portion of their leaves has long been treated as an important component of drought adaptation (Santiago et al., 2004; Hasselquist et al., 2010), which could reduce their water demand during drought phase and facilitate their recovery after drought. As extreme drought will becoming more frequent in the study area due to climate change (Liu et al., 2015), water conditions for certain habitat types may become more unpredictable; the advantages to having a plastic root system may be enhanced by the associated drought adaptation.

## Conclusion

In order to determine the degree to which shallow soil-adapted species explore and rely on rock fractures and whether drought stress has strong effects on root deployment, three tree species that are native to a typical shallow soil region (a karst region in southwest China) were selected based on the expectation that there would be a relationship between seedling growth rate and root exploration of rock fractures. Our results revealed diverse rooting strategies that were employed by shallow soil-adapted species, however, they were more closely related to the species-specific drought adaptation rather than growth rate. Rooting strategies that are efficient in fracture exploration are not a necessarily a speciality of shallow soil-adapted species; repeated cycles of drought stress only caused additional effects on root deployment of one of the three species. The success of these species in shallow soils should benefit from the mutual complementation between their species-specific rooting strategies and drought adaptations. On the other hand, this diverse rooting strategies, as well as the associated species-specific drought adaptation, was likely the key reason for the coexistence of these different tree species in shallow soil systems. As climate extremes are becoming more frequent and unpredictable with global climate change, the adaptation of these strategies to different climate scenarios needs to be tested with further studies.

## Author Contributions

YN and HC designed the study, performed the experiment, collected and analyzed the data, and wrote the manuscript. YD, JY, and KW discussed the experiment design and manuscript writing.

## Conflict of Interest Statement

The authors declare that the research was conducted in the absence of any commercial or financial relationships that could be construed as a potential conflict of interest.
